# Factors affecting the well-being of patients with thyroid cancer: results of a UK qualitative study

**DOI:** 10.1136/bmjopen-2025-099254

**Published:** 2025-10-23

**Authors:** Alicja Rogusz, Jamie Harding

**Affiliations:** 1 Faculty of Health Sciences and Wellbeing, University of Sunderland, Sunderland, UK; 2 Department of Social Sciences, Northumbria University, Newcastle upon Tyne, UK

**Keywords:** Adult oncology, Quality of Life, Thyroid disease, Nursing Care, Patient-Centered Care, Patient Satisfaction

## Abstract

**Abstract:**

**Objectives:**

The study sought to understand the experiences of patients with thyroid cancer (TC) from their own perspective and to identify the factors that affected their well-being.

**Design:**

This was a qualitative study based on semistructured interviews that collected both prospective and retrospective longitudinal data.

**Setting:**

Patients were initially recruited from one National Health Service (NHS) Trust and from the contact list of a TC charity.

**Participants:**

25 participants took part in the study: 22 female and 3 male. The inclusion criteria were: (1) adults over 18 years of age; (2) patients diagnosed with papillary or follicular TC within 5 years of diagnosis and (3) patients able to give informed consent. The exclusion criteria were: (1) diagnosis of anaplastic TC; (2) diagnosis of terminal TC with a short life span prediction and (3) codiagnosis of another condition in addition to TC.

**Results:**

Patients’ psychological health, physical health, relationships, employment and finances are all likely to be affected by the diagnosis and treatment of TC. Negative factors that affect the overall experience can include a lack of compassion from healthcare professionals, as well as physical side effects after surgery and during recovery. Isolation and loneliness can be significant at many stages, most frequently during treatment with radioactive iodine. Anxiety and fear were widespread among participants.

**Conclusions:**

The experiences of TC patients can be challenging, with well-being influenced by treatment effects, psychological distress and the quality of support available. These findings suggest that enhanced patient education, emotional support and follow-up care may help improve well-being, although further research is needed to explore how best to implement such approaches.

**STRENGTHS AND LIMITATIONS OF THIS STUDY**

- Use of qualitative interviews allowed an in-depth exploration of patient experiences.
- Semistructured interviews provided flexibility to capture both anticipated and unanticipated aspects of the cancer journey.
- A combination of prospective and retrospective interviews offered breadth, but recall bias may have influenced retrospective accounts.
- The data were based on interviews with 25 patients who were unlikely to be representative of all patients with thyroid cancer.
- Recruitment challenges meant the original prospective longitudinal design was modified, which limited the ability to capture experiences in real time.

## Introduction

 Thyroid cancer (TC) is the 20th most common form of cancer in the UK, and its incidence is rising; there were 3865 new cases reported annually between 2016 and 2018, translating to an average of 11 new cases per day.^1^ The mortality rate for TC is relatively low, and there is a growing number of survivors, with a 10-year survival rate of 85%.^1^

In the diagnosis stage, patients may undergo various clinical examinations, including examination of the vocal cords with an endoscope, palpation of neck and lymph nodes, blood tests, ultrasound (US) and biopsy.^2^ The treatment may include partial thyroidectomy (PT), total thyroidectomy (TT), neck dissection, lobectomy, radioactive iodine (RAI) treatment and active surveillance. Some patients have to have a second hemithyroidectomy. The treatment journey of people with TCs can be complex and lengthy. However, new approaches to diagnosis and treatment are being developed, moving towards less invasive testing and treatment and increased use of active surveillance.^3^

Previous research has suggested that the factors needed to maintain a good prognosis are accurate diagnosis, appropriate treatment and long-term regular follow-ups.^4 5^ In patients with classical, well-differentiated TC, very aggressive forms can occasionally be observed, making well-differentiated TC, in some cases, unpredictable.^6^ Patients who have a history of TC, particularly micropapillary cancer, are at higher risk of developing different forms of cancer.^7^ Metastases and the recurrence of the disease can happen even decades after the initial diagnosis. For this reason, some TC patients may require ongoing follow-up for many years.^5^

TC treatments are associated with a wide range of side effects that can significantly affect long-term quality of life. Surgery may lead to recurrent laryngeal nerve palsy, dysphagia, scarring, hypoparathyroidism or neck discomfort, with voice changes and mobility limitations strongly linked to impaired well-being.^8^,^12^ RAI therapy can cause salivary gland dysfunction, taste alterations, xerostomia and chronic oral complications, with symptoms persisting in a subset of patients.^13^,^15^ Hormone suppression therapy may produce symptoms of both hypothyroidism and hyperthyroidism, including fatigue, weight changes, cardiovascular risks and cognitive difficulties such as memory loss and impaired concentration.^16 17^ These side effects can significantly impact the quality of life,^18 19^ even many years after diagnosis.^20 21^

Existing research on patient experiences has primarily relied on quantitative methods.^22 23^ The small number of qualitative studies that exist has been conducted in the USA,^24 25^ South Korea,^26^ Canada^27^ and Australia.^28^ The study discussed here is, therefore, the first one to use qualitative methods to explore UK patients’ experience and well-being from their own perspective.

Subjective well-being is a dimension of psychological well-being that consists of people’s own evaluation of their lives. It is often omitted when well-being is measured by measures other than self-reports.^29^ The subjective well-being framework has two layers: cognitive and emotional. Cognitive incorporates life satisfaction, and emotional describes the presence or absence of positive or negative emotions and thoughts. It can also include feelings and reflection.^30^ The 5S Framework of well-being is one of the more comprehensive approaches to looking at areas of life that impact a person’s subjective well-being.^31^ These frameworks will be used when discussing the results.

### Objectives of the study

The first objective of the study was to use qualitative methods to explore the experiences of TC patients, from diagnosis to medical recovery and beyond, from the patient’s perspective. The second was to explore the impact of hospital care and treatment. The third was to establish which factors affected the lived experience and well-being in a positive and negative way. The fourth was to develop practice recommendations based on insights gained from patient narratives.

## Materials and methods

### Design

Qualitative descriptive phenomenology was the methodology used in this study. The paradigm was interpretivism, which is consistent with the subjective accounts of participants that were gathered. The Standard for Reporting Qualitative Research Checklist was used to ensure the rigour and transparency of reporting.

### Setting, participants and sampling

The study used purposive sampling methods. Patients were initially recruited from an NHS TC clinic setting, with the help of a local collaborator, and later through a social media appeal by a relevant registered charity: an approach that had been approved through the ethics process. For patients recruited from the clinic, information packs were provided about the study. Interested patients contacted the researcher, and the participant information sheet (PIS) and consent form were then sent out. In the case of the patients recruited by the charity, an advert was posted on social media, inviting potential participants to contact the researchers.

The participants were from a variety of UK locations. The inclusion criteria were (1) adults over 18 years of age; (2) patients diagnosed with papillary or follicular TC within 5 years of diagnosis; (3) able to give informed consent. The exclusion criteria were (1) diagnosis of anaplastic TC; (2) diagnosis of terminal TC with short life span prediction and (3) codiagnosis of other cancers than TC.

Determining the sample size that is adequate for a qualitative study is a difficult issue. Attempts to determine a minimum sample size using fixed, a priori methods are fundamentally flawed.^32^ The use of the concept of saturation would also have been problematic in this case, as it was originally developed only for use in grounded theory studies.^33^ Thus, the authors adopted the view promoted by qualitative methods specialists^34^ that the more information the sample holds, relevant for the actual study, the lower the number of participants needed. It is the contention of the authors that the large amounts of information provided by the sample, as demonstrated by the discussion below, make the sample size of 25 more than adequate.

### Data collection

A questionnaire was used for demographic and clinical information, then a semistructured interview covered the TC journey chronologically by asking about the following areas: (a) life before diagnosis; (b) experience of diagnosis; (c) experience of treatments and (d) experience of life after treatment and follow-up. The interview guide (online supplemental file 1) was refined iteratively: it was first tested with one TC survivor and adjusted based on that interview. As further interviews were conducted, the researcher continuously adapted the sequence and phrasing of questions, so the guide evolved throughout the study rather than being formally piloted in advance. The interviews were intended to look at participants’ lives holistically without the limitation of, for example, looking at health-related quality of life only. The interview guide is attached; all interviews were conducted by one of the authors (AR).

All participants provided written informed consent after reading the PIS. Data were collected in 2019 and 2020: the face-to-face method of interviewing had to be modified to online interviewing after the outbreak of COVID-19 (ethical approval was granted for this change). No compensation was offered to participants.

The original intention for the data collection was to interview patients shortly after diagnosis, 6 months after diagnosis and 1 year after diagnosis. The rationale was to record contemporaneously the subjective nature of the experience at different stages of their cancer journey. However, when recruitment proved insufficient by this method, an alternative approach was adopted of recruiting patients through the TC charity—these were all patients who had completed their treatment and so discussed different stages of the process retrospectively. However, their recall was strong and vivid, meaning that the researchers were confident that the hybrid longitudinal approach had provided a clear picture of the chronology of patients’ experiences.

### Data analysis

**Figure 1:**
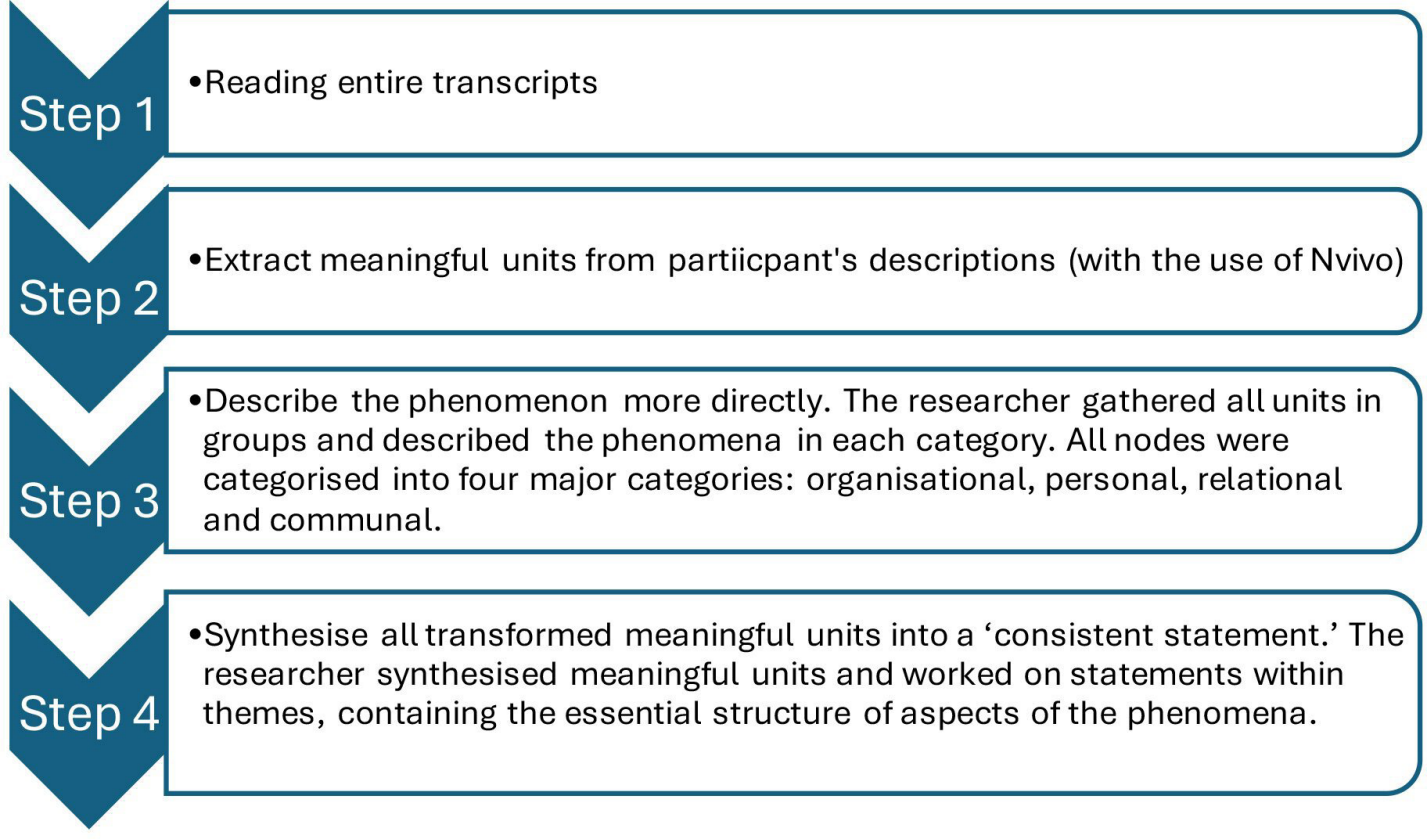
Descriptive phenomenology analysis steps. This figure outlines the four key stages of the descriptive phenomenology analysis process. Step 1 involved reading the entire transcripts. Step 2 entailed extracting meaningful units from participants’ descriptions using NVivo. In step 3, the researcher grouped units and described the phenomena within categories (organisational, personal, relational and communal). Step 4 synthesised transformed meaningful units into a consistent statement, reflecting the essential structure of the phenomena.

Interviews were recorded, transcribed verbatim and analysed using Georgi’s descriptive phenomenology analysis^35^ and content analysis. The conceptual frameworks of subjective well-being^29^ and the 5S framework of well-being,^31^ discussed above, were used to identify the factors that impact subjective well-being. NVivo V.12 was used for coding in step 2 of the analytical process in thematic analysis. Georgi’s analysis method process is shown in figure 1. Content analysis was manual, using a table in Microsoft Word. All the transcripts were read, and key factors were identified. Then the researcher established whether the factors were addressed by all participants.

### Assuring rigour

To ensure trustworthiness of the study, steps were taken to ensure dependability, credibility, transferability and conformability of the findings. To ascertain dependability and credibility, a rigorous methodology was used throughout the study. The first author, AR, has a nursing clinical background as well as a clinical research nurse background, but was not involved in the care of the participants. Moreover, the author received extensive research training through the National Institute for Health and Care Research (NIHR), including the GCP. To aid credibility, two data analysis techniques were used (triangulation of analysis). The second researcher, JH, looked at five of the transcripts to ensure similar themes were identified, in order to further enhance credibility (analyst triangulations). This process demonstrated a high level of agreement about the findings that were emerging. To ensure confirmability, the researcher used reflexivity when collecting and analysing data to avoid impacting the process with previous knowledge and assumptions, as well as experiences regarding the disease. Transferability was met by a detailed description of the participants and setting so that the reader can decide on the relevance of the study.

### Patient and public involvement and engagement

The researcher sought patient and public involvement early in the design of the study. Questions were asked regarding the readability, volubility and reasonableness of the study documents, such as the PIS and interview guide. These discussions helped with recruitment, study design and data collection. The people consulted were (1) two patients with TC, one current and one survivor of TC; (2) TC Medical Clinicians from National Health Service (NHS) Trust; (3) an NHS TC Nurse Specialist; (4) Staff of Butterfly Thyroid Cancer Trust (BTCT) and (5) a research nurse from an NIHR CRN.

## Results

### Characteristics of study participants

Of the 25 participants, 8 were recruited within 8 weeks of diagnosis and were interviewed up to 3 times through different phases of treatment. Others, who were recruited within 4 years of diagnosis (n=17), undertook a single, retrospective interview after the treatment was completed. Altogether, there were 33 interviews conducted and 1 email update. This information is presented in table 1.

**Table 1:** Recruited participants’ source and codes

NHS-recruited prospective participants (within 8 weeks of diagnosis)	3 (2 participants had 3 interviews, 1 participant had 2 interviews and 1 email update) NHS01-NHS03	NHS01-NHS03
NHS-recruited retrospective participants (within 4 years of diagnosis)	0	
Non-NHS-recruited prospective participants (within 8 weeks of diagnosis),	5 (1 participant had only 1 interview, 2 participants had 2 interviews and 1 participant had all 3 interviews).	TC01-TC22
Non-NHS-recruited retrospective participants (within 4 years of diagnosis)	17 (1 interview per participant)	

NHS, National Health Service.

Most participants were female, of working age, employed, married, had children and had no significant medical history (table 2). The majority were diagnosed with papillary TC and had a TT or PT and RAI treatment (table 3). All participants were within 4 years of diagnosis.

**Table 2:** Sociodemographic characteristics

Sociodemographic characteristics	Frequency	Percentage
Gender		
Female	22	88
Male	3	12
Other	0	0
Age		
18–30	4	16
30–50	11	44
50–65	9	36
Over 65	1	4
Marital		
Married	15	60
In relationship	3	12
Single	3	12
Engaged	2	8
Divorced	2	8
Children	8	32
No children	6	24
Adult children	4	16
Young children	4	16
Teen children	2	8
Pregnant	1	4
Living situation		
Living with husband/partner and kids	11	44
Living with husband/wife	6	24
Living alone	4	16
Living with partner	3	12
Living with parents	1	4
Employment		
Full-time	9	36
Retired	6	24
Self-employed	4	16
Part-time	3	12
Full-time study	2	8
Homemaker	1	4

**Table 3:** Clinical characteristics

Treatment	Frequency	Percentage
PTC (papillary TC)	15	60
FTC (follicular TC)	5	20
Met PTC (metastatic PTC)	2	8
Met FTC (metastatic FTC)	1	4
PTC and Hürthle cell TC	1	4
PTC FTC and Hürthle	1	4
Diagnosis		
PT then second PT and RAI treatment	9	36
TT, neck dissection and RAI	7	28
TT and RAI	3	12
TT	3	12
PT	2	8
PT, then second PT	1	4
Medical history		
None	14	56
Type 2 diabetes mellitus	2	8
Depression	3	12
Other	11	44

PT, partial thyroidectomy; RAI, radioactive iodine; TC, thyroid cancer; TT, total thyroidectomy.

### Results of content analysis

#### Factors affecting the subjective well-being framework

**Figure 2:**
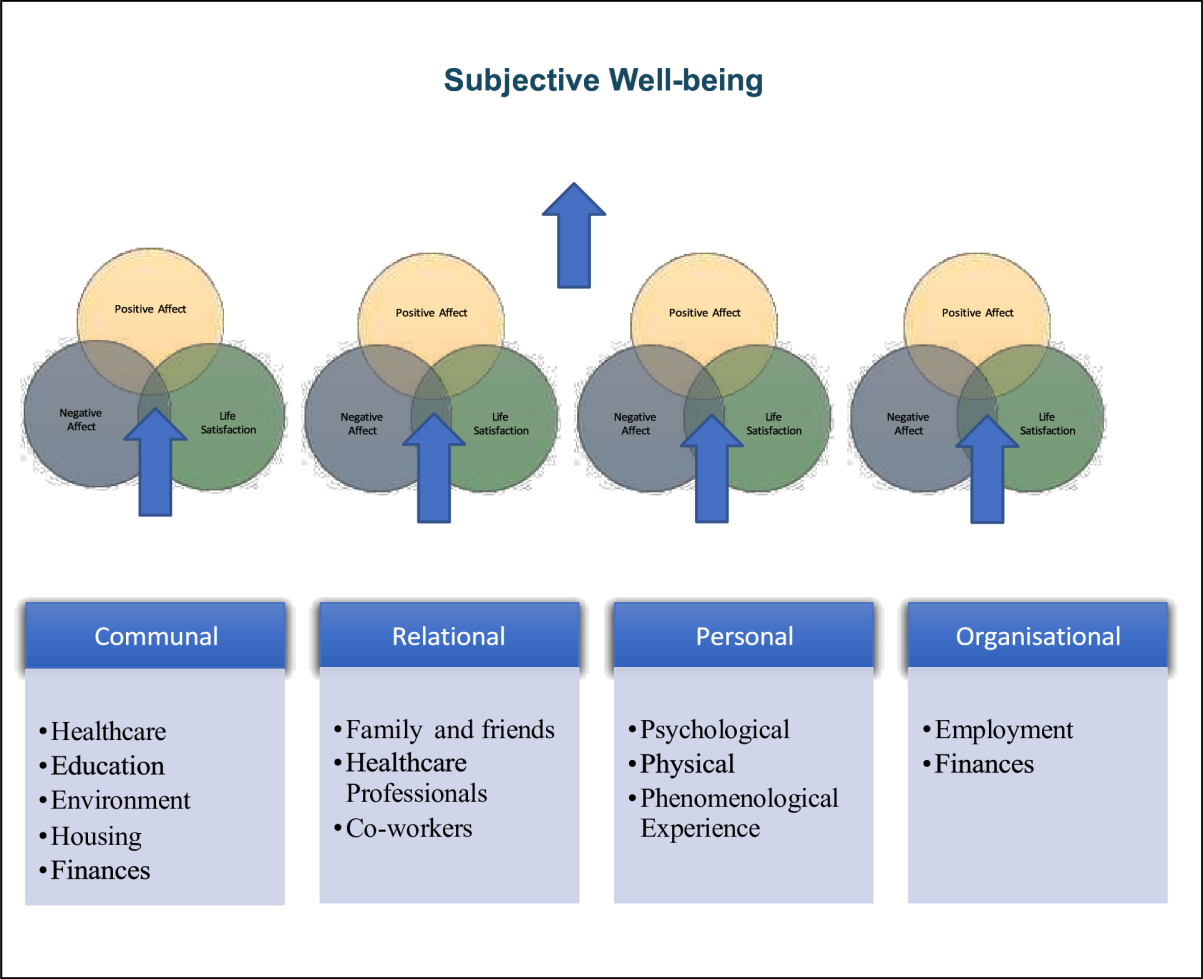
Conceptual framework of subjective well-being in people with TC. This figure illustrates how factors from different life domains influence the three components of subjective well-being: positive affect, negative affect and life satisfaction. The framework includes communal factors (healthcare, education, environment, housing, finances), relational factors (family and friends, healthcare professionals, coworkers), personal factors (psychological, physical, phenomenological experience) and organisational factors (employment, finances). TC, thyroid cancer.

The 5S framework and the concept of subjective well-being were used in combination to present the factors affecting well-being from the content analysis. As shown in figure 2, factors from different domains of life impact negative/positive emotions and life satisfaction, which are the components of subjective well-being.

#### Factors that impact subjective well-being

As presented in table 4, the most consistently reported positive factors across all participants included support from family and friends, satisfaction with surgical scars, access to clear information, the presence of a supportive partner, encouragement from healthcare professionals (HCPs) and charities, positive surgical outcomes and effective coping strategies. In contrast, the most commonly identified negative factors were long-term physical symptoms, ongoing psychological difficulties, fatigue and postoperative complications. It is important to note that patients sometimes assessed distinct elements of the journey differently—for example, some were satisfied with the information provided at some stages but not at others.

**Table 4:** Negative and positive factors affecting well-being

Positive factors	Out of 25	Negative factors	Out of 25
	Communal	Factors	
Positive experience of surgery	19	Negative experience of recovery	13
Well informed	19	Complains about NHS	10
Happy with monitoring	17	Negative experience with COVID-19	9
Positive recovery	13	Negative experience of RAI	8
Positive experience of RAI	11	Negative experience of surgery	8
No complains about NHS	11	Not well-informed	7
Positive experience related to COVID-19	6	Negative second surgery	4
Positive experience of second surgery	4	Unhappy about monitoring	4
	Relational	Factors	
Family supportive	23	No need for support	11
Partner supportive	19	No charity support	9
HCP supportive	19	No HCP support	6
Friends supportive	15	No life partner or not supportive	5
Accessed charity support	15		
Work supportive	13		
	Psychological	Factors (Personal)	
Coping well	16	Psychological symptoms	19
Psychological health improves over	12	Distress waiting	13
Psychological health ok	9	No coping well	11
Coming to terms with a diagnosis	9	Not coming to terms with the diagnosis	5
	Physical	Factors (personal)	
Satisfied with scar	23	Long term physical symptoms	21
No fatigue	5		
Positive changes to fitness	4	Fatigue	19
		Postoperative symptoms	17
		Negative changes to fitness	3
		Dissatisfied with scar	3
	Organisational		
Life is back to normal	9	Work affected	12
		Finances affected	10

HCP, healthcare professional; NHS, National Health Service; RAI, radioactive iodine.

### Results of thematic analysis

Three main themes were identified: (1) the experience of TC treatment and care, (2) mental health effects of treatments TC treatment and (3) life as a survivor (table 5).

**Table 5:** Thematic analysis

Theme 1. The experience of TC treatment and care	The experience of TC: the diagnosis
	Experience of TC: the surgery
	Experience of TC: the RAI therapy
	Experience of TC: the support
	Experience of TC: information provision
	Experience of TC: treatment during COVID-19
Theme 2. Mental health effects of TC treatment	Mental health effects of TC
Theme 3. Life as a survivor	Life as a survivor: long-term physical side effects
	Life as a survivor: moving on
	Life as a survivor: work and finances

RAI, radioactive iodine; TC, thyroid cancer.

### The experience of TC: the diagnosis

Most participants were healthy, active and had a healthy diet before diagnosis. Their lives were busy with work, family life and keeping fit. Being active and healthy were the most significant emerging theme when talking about life before being diagnosed with TC. Most participants found the lump accidentally, did not know much about TC at that time and were in shock after the discovery. The waiting period before diagnosis was very distressing for many:

This is why one of the problems with well-being and thyroid cancer patients is so bad, it takes a long time between when they get a diagnosis and when they receive the treatment. NHS02int1 (Participant NHS02, first interview)

Moreover, many participants had anxiety regarding surgery, possible side effects and about having scars on their neck:

I was scared, they told me I would have an operation on my neck, and that I would have a scar on my neck, so that really frightened me, as a woman, you are more conscious about your body, so I had to think ok, now I will have a big scar on my body. TC11

At these early stages of diagnosis, family support and the attitude of HCPs were very impactful on the patients’ journey. According to participants, being well-informed and reassured by a trustworthy healthcare team had a positive impact on their well-being in the early stages.

### Experience of TC: the surgery

The majority of patients experienced excellent care in the hospital. This was mainly due to the care provided by HCPs:

It was amazing, I was in awe of the NHS before but after this and obviously after lockdown, they couldn’t be more reassuring, very personal. I was lucky that the hospital I went to had a new treatment centre. Couldn’t be more reassuring. (…) I can’t fault my care at the hospital at all. TC15

Participants valued medical care as well as good preoperative and postoperative nursing care. Care was described by study participants as ‘superb’, ‘lovely’, ‘great’, ‘kind’, ‘excellent’, ‘brilliant’, ‘good guys’, ‘a top surgeon’ and ‘attentive’. Most participants felt that HCPs were informative and supportive, they could ask for help at any point, and that they were treated with ‘respect’, ‘dignity’, ‘care’ and ‘attention’. One participant felt that the kindness of everyone in the hospital made it a positive experience.

Negative experiences of surgery were related to high levels of pain and discomfort, poor experiences of care, lack of adequate support and a disturbing environment. The most common physical complaints were tiredness, pain, swelling, voice problems and swallowing discomfort:

When I woke up, I didn’t feel very well. I was in a lot of pain, actually, my experience of being in the hospital wasn’t very good. And I was waiting very long for pain medication. I think sometimes I was waiting up to 2 hours for pain medication. I don’t think that the level of care was up to standard. I appreciate NHS staff are stretched, but at a minimum, you would expect a level of care. At night, I was on my own; it is not that my family were there to comfort me. I was on my own, and I was in pain, and the strongest pain medication was paracetamol. Yes, they gave me paracetamol and ibuprofen. And because it was a neck dissection, it went all the way around my neck, that is where it was very painful and swollen. TC11

Although some participants were distressed to be told that they needed a second surgery, the surgery itself tended not to significantly impact people as most knew what to expect and had a better experience than with the first surgery. Only two study participants had a single hemithyroidectomy as their treatment, without needing a second operation.

From an organisational perspective, around half of the participants in the study were happy with the management of their disease through the healthcare system. However, others had complaints regarding waiting times between treatments, the treatment being disjointed due to the involvement of many clinical teams, operations being wrongly conducted and cancelled appointments and surgeries.

### Experience of TC: the RAI therapy

RAI treatment was a positive or neutral experience for many participants interviewed in the study:

Staff were very good, and they said we know you are on your own here, but we are here if you need anything. And they kept sticking their heads round the door saying “you ok”? Even if they were doing it through the night. But I know they were just doing their job. The hospital I was at is very good, the food was good, the staff are good. I didn’t feel any sickness or anything. I was quite surprised that I could go home so quickly. TC01int2

However, some participants thought it was very challenging, especially the loneliness and isolation that were involved

It was a horrible experience being shut in that room, with no contact with anyone. I had a telephone, you really did feel unwanted, unloved. I went on Wednesday till Friday. The second time was the same. The second time was nicer because the room was decorated. At the first time I think the prisoners have better rooms. First time round I had to phone them to bring me a jug of water, because they forgot all about me. TC05

Other difficulties identified by participants were feeling forgotten, issues with hospital food and boredom. In addition, while most people did not experience significant physical symptoms during or following RAI treatment, some felt pain in their salivary glands, tiredness and temporary taste alterations.

### Experience of TC: the support

Participants sought and utilised various support tools to help them get through the disease and treatment. The main form of support was counselling, therapy, materials provided by charities and Facebook groups:

The DVD from Butterfly was amazing, but I should have received it at the beginning not after my whole treatment finished and only after me googling and finding butterfly. TC04

A minority of people accessed other professionals to support them with physical symptoms or a psychologist or GP to support them with psychological symptoms. People may not access support because they are not aware that it is available. Alternatively, they may fear that they will not be able to relate to people with other forms of cancer or older cancer patients. Some participants stated that they could not justify seeking support in their position:

I mean it is quite tricky because, in some ways, it is like I am healthy and I don’t really want to bother people, maybe they are busy dealing with people with more aggressive forms of thyroid cancer, I just kind of think I don’t want to trouble them. NHS02int3

An important aspect of the TC journey is support from friends and family. Most participants felt well supported, but for some, TC initiated the breakdown of the relationship with a partner, while others experienced some challenges in their relationships:

Yes, there is a strain there, because there is a lot of worry on both sides. And at times I think that we both can be a little bit short tempered. We are identifying when it is happening, and we are trying to resolve it, but we have noticed that once or twice, both of us will be a little bit short tempered with each other. We don’t fall out. You know, it starts to happen, we bring it down again. There is obviously stress in there, which you can notice (…). NHS01int1

A TC diagnosis and treatment are also challenging for family members, inducing stress and anxiety. Apart from family support, another important social source of support was friends. Relationships with friends were supportive for most participants:

I’ve got friends from work, they were very supportive, we are sort of the same age, and we are a very small group. They supported me, and I have some friends in another part of the country and they stood behind me 100%. So, yes, I felt supported by my friends and colleagues and that is what got me through it. TC16

However, a small number of participants mentioned that some of their friends could not seem to handle their cancer diagnosis:

Something I noticed with people, not everyone was able to ask me how I was, some people just completely disappeared from my life, it is like they couldn’t handle it. Another thing was that people thought that, after my RAI was over, they just thought it is done, it is over, they don’t realise that this is ongoing. I don’t receive any questions about how I am doing, and it actually hurts a bit, it is still ongoing, it is still happening. TC09

### Experience of TC: the information provision

Information provision is a topic that was often mentioned by study participants and some gaps were identified. Some participants said they found information that they were provided with too general and not TC-specific. A common complaint was a lack of information about taking levothyroxine. Some participants mentioned that they were not prepared to deal with physiotherapy and exercises. Other information gaps discussed were how unwell they would feel after the operation, swallowing after the operation, stiffness and the length of follow-ups.

They told me that it is slow growing and I will be fine. They played it down a lot. I don’t think they prepared me for all the side effects, apart from the trauma of the operation I was not ready and prepared for the symptoms and side effects. TC09

### Experience of TC: the treatment during COVID-19

Multiple individuals involved in this study underwent surgeries amid the COVID-19 pandemic. Those who received treatment during this time were required to quarantine for 2 weeks prior to their procedures and for 1 week afterwards. They reported a variety of challenges, such as being released early, the need for isolation, instructions to attend alone and difficulties communicating with staff dressed in personal protective equipment. Two participants noted that the overall experience felt surreal because of the infection control protocols in place at the hospital:

The second time was a little bit like an out of body experience, the only way I could go through it was to detach myself from what was happening. TC03int1

One major concern was isolation; restrictions meant that visitors were not allowed, which added to the distress of many. However, on a more positive note, some participants also highlighted advantages of receiving treatment during this time:

Maybe it was a blessing in disguise as I did enjoy my retirement, the coronavirus was a bit of a blessing for me, because when I was off my treatment, I felt brilliant, I had lots of energy I was able to do lots of things in my garden. TC05

### Mental health effects of TC treatment

The psychological health of patients with TC was significantly affected, although most symptoms improved over time. Anxiety symptoms and low mood were the most common:

You know, when you have your thyroid removed, thyroid regulates all your body organs, so it actually affects your whole mood. And people who have underactive thyroid find that they have problems with mood. You know, and I do have days when I have low mood. (…). I’ve never had any problems with low mood before. I’ve always been happy and optimistic. I must say that since having this diagnosis. NHS02int1

Participants of this study were worried about recurrence, metastasis, other unrelated cancers and long-term physical symptoms:

I think the worry that it may come back is the big one, because you have so many tests all the time. (…) Some people say “oh it is thyroid cancer, you will be fine, oh, it’s cancer-like, it is not a cancer”. And I think that is very unfair, because it still kills people. And it had a big impact on my life. TC10

When relevant, participants were worried about their future fertility. Follow-up appointments and the need to be monitored for the rest of their lives could be both reassuring and worrying.

Following the treatment, participants described the process of accepting that the TC experience is part of their lives and moving on. The anxiety often improved with time:

I did not want to be stuck; my anxiety was horrendous, mentally, I am a 100% better. In all fairness, even before I found out about cancer and compared to now, mentally, I am in a better place. I don’t know, my whole mindset has changed, but the CBT has definitely helped, just knowing that I will be ok, and I am moving forward. I don’t want to be stuck in the past, and thinking about it and talking about it. TC17int3

### Life as a survivor: long-term symptoms

Following the treatment, many long-term physical symptoms can impact survivors’ lives. Fatigue, neck pain and tightness, voice problems, permanent swelling and heart palpitations are among the symptoms mentioned by the study participants:

And I do still have fatigue. I don’t have the energy that I used to have. But apart from that, I don’t have any other symptoms really, (…). But because I am still quite fatigued, I kind of feel that I lack the energy that I have; I kind of lack that spark for life that I used to have. NHS02int3

Most participants were cleared of the disease when the study was conducted. However, some participants had metastasis of their TC. Naturally, this provides even more worry and anxiety for participants, as well as necessitating further treatments and monitoring, which made it impossible to move on from the disease.

### Life as a survivor: moving on

Many described processes of transformation following their treatment for TC. Three main areas of positive change were a more positive and appreciative attitude toward life and other people, adopting healthier lifestyle habits to improve health and actively helping others through fundraising, volunteering and participating in TC-related research:

But it also changed me in a good way. I feel more appreciative. I know it sounds cheesy, but it just makes you appreciate more, and it puts into perspective what is important in life. For me, being happy and healthy is important, but sometimes they are things out of your control. I think from that point of view, it made me work harder on being happier and spending time with friends and family when I can. I don’t know, I feel different in a sense that I have grown, become more mature. I think when you experience something like that, you know, shocking and life-changing it just means that maybe you are more prepared for things. I think if in the future something major happens, I will be more prepared for it, because I had this experience. TC11

### Life as a survivor: work and finances

Work and finances are other areas heavily affected by the disease and treatment. Key requirements for participants were that the workplace was flexible, agreed to time off for the appointments and was supportive/flexible with substantial sick leave due to a long recovery time. Difficulties could be caused by unsupportive managers who did not recognise the need for time off for appointments or for surgery and recovery. TC can impact a patient’s working life, career trajectory and financial situation, leading to further difficulties:

There was a lot of worry when, after 6 weeks, I handed my notice in, that was the point when everything caught up with me, we lost financially overnight, one wage completely, feeling poorly with migraines and psychologically, and to be fair, I had a little nervous breakdown. TC22

However, some participants had supportive employers and had not experienced significant changes in their working lives due to TC.

They were extremely supportive. I told them what it was, they couldn’t be more supportive. They worked by me, if I said I had to work half a day or start later or finish later, they were fantastic. TC15

As seen above, participants had a range of comments—positive, neutral and negative—about their experiences. They presented both negative and positive emotions that impact their subjective well-being.

## Discussion of key results

There is a notable gap in the published literature regarding the subjective experiences of patients undergoing treatment for TC within the UK. Specifically, insights pertaining to patient experiences in hospital care settings remain significantly underexplored. Most existing studies predominantly focus on quantifying the effects of treatment and its subsequent impact on quality of life. This study represents a significant contribution to the existing body of knowledge regarding the experiences of patients diagnosed with TC within the UK context. The outcomes of this research enrich the understanding of the multifaceted experiences encountered by these patients, highlighting critical aspects that may influence their overall well-being. Furthermore, the findings have the potential to inform the formulation of targeted interventions aimed at mitigating the adverse factors that can negatively affect patients’ experiences. The research’s methodological design, which included participants recruited from diverse geographical locations across the UK, enhances the transferability and applicability of the findings to various clinical settings throughout the country.

The strength of this study lies in its comprehensive identification of a diverse array of factors influencing the well-being of patients diagnosed with TC, which helps to explain why patients may experience diminished quality of life, despite their relatively favourable prognostic outlook. Utilising a qualitative methodology, the research afforded patients the opportunity to articulate the factors they deemed significant, thereby providing nuanced perspectives on their lived experience and their subjective well-being. The principal findings are categorised into four key areas: care and long-term side effects; support and provision of information; mental health; and employment and financial considerations.

### Care and short-term and long-term side effects of treatment

Most participants experienced excellent care in the hospital, including positive interactions with HCPs. However, it is important to note here that a few participants did not feel treated well at some stages and felt that their relationships with HCPs were poor. Previous studies have shown that the extent to which patients have trusting relationships with HCPs can have a major impact on quality of life,^14 36^ and with that, on well-being.

Eight participants (32%) in this study experienced postoperative complications. The most common complication was neck swelling that needed to be drained. Other complications included wound infection, calcium deficiency issues, hypertension, shoulder nerve damage, seizures caused by low calcium and permanent facial swelling. Previous research has demonstrated that the quality of surgery impacts overall well-being,^22 37^ especially when low-quality surgery leads to side effects or complications.^38^ Two participants experienced major mistakes in their surgeries and had the wrong side of the thyroid removed. This is very rarely reported in the literature^39^ and could be an effect of self-selection bias. While complications are relatively common, serious mistakes during operation are rare, but have major implications for well-being.

Although some side effects of the treatment were short-lived, 21 of 25 participants reported long-term symptoms. The most common of these was fatigue—a symptom frequently noted in other studies^1840^,^44^—followed by neck tightness. Other reported long-term side effects included voice problems, swelling, dry skin and heart palpitations. There were fewer reports of some of the long-term symptoms described in other studies, such as memory problems,^45 46^ headaches and psychological issues.^14 46^ However, although there were no reports of headaches or memory problems, a few participants reported a brain fog phenomenon and distressing difficulty coping in the work environment. It is important to note that this study included patients only up to 4 years of diagnosis, so the longer-term effects of TC treatment go beyond its parameters.

This study also noted some specific side effects of the RAI part of the TC treatment that affected the well-being of patients. Although it echoed the findings of previous studies^23^ that these side effects were usually short-term, their impact could be quite severe. In addition to fatigue, the most common side effects were taste alterations and sore salivary glands. The pain from the salivary glands could produce a high level of discomfort, as has been noted elsewhere.^14 15 46^ One participant of this study thought that there may be a marginal permanent difference in their taste sensation, and another felt occasional tightness in their salivary glands, but only when dehydrated.

### Support and information provision

While most participants had extremely helpful and supportive personal relationships, there were reports of a lack of understanding among some family and friends, who did not think their condition was serious or became more focused on their own distress than that of the patient. These findings were consistent with those of other studies showing that the quality of relationships can have a substantial impact on the well-being of patients.^13 19 47 48^

Having accurate information could be central to meeting emotional needs and dealing with fears/anxieties. However, despite its widely acknowledged importance to the well-being of patients with TC,^17 49^ some respondents were dissatisfied with the information they received, the stage of the process at which they received it and/or its relevance to TC patients. For example, some received information about general thyroidectomy that didn’t address their specific concerns and seemed inappropriate. In contrast, the more tailored information provided by BTCT and Macmillan was particularly appreciated.

### Mental health

Several participants in this study reported increases in symptoms of anxiety and depression after getting their diagnosis. This is a factor that is frequently noted in the literature regarding cancer in general^50^,^53^ and TC in particular.^8 23^ Symptoms of anxiety and depression could become evident at any phase of treatment; another finding that is consistent with previous research.^15^ Factors that were particularly likely to cause worry or concern for participants in this study were future prognosis, recurrence, future metastasis and getting diagnosed with other cancers.^14 48^ Many participants of this study thought frequently about their own mortality and death.^14 54^ The waits for diagnosis and treatment were particularly worrying times, and side effects were a constant fear.^13 42 54^ Fears could be heightened by reading stories online of people who had had negative experiences. These fears made the support of HCPs particularly important, but some participants said that HCPs did not mention or acknowledge the emotional side of the process. People with TC have many fears and anxieties, which can directly impact their subjective well-being.

RAI could also have a detrimental impact on the feelings and well-being of the participants, another finding that is consistent with the existing literature in this area.^17 43 55 56^ As noted above, the factors that participants felt had negatively impacted their mental health and well-being during the treatment were related to isolation, going to the hospital alone (during COVID-19), feeling forgotten about, confinement, being bored, having to remain within a small suite and being dissatisfied with the food. The substantial impact of isolation has also been highlighted by previous studies.^44 45^ Sometimes participants had to stay away from their children even after leaving the hospital, intensifying the distress. Some participants welcomed the RAI therapy time, with one participant welcoming it as a break from busy family life.

### Work and finances

TC mainly affects younger people, and 64% were in some form of employment at diagnosis—a similar figure to one found in previous research.^46^ The TC diagnosis and treatment could have employment implications, which included making changes to a patient’s role, missing out on promotions and career advancements, prolonging full-time study and taking early retirement. A Dutch study^57^ showed a similar range of impacts, with the addition that some participants were dismissed from their employment. Although no participants of this study were forced to leave employment, two gave up their jobs following their treatment: one said that their manager did not regard surgery on their neck as a serious medical issue, while another said that struggles and stresses around work were the most significant and challenging issues affecting their well-being during the TC journey. Other studies have included examples of survivors who did not return to work following a TC diagnosis.^58^

Previous studies have shown that financial difficulties are more common among TC patients than patients with other malignancies.^59^ This seems likely to be explained by the majority of patients being of working age. The link between financial situation and well-being is well established,^29 60^ and some previous studies have shown that financial situation can add to the emotional and physical burden of TC patients,^15 61^ although other research has contradicted this finding.^16^ In this study, a small number of participants found that their financial situation, which changed due to TC, had a negative impact on their well-being.

### Limitations

The main weakness of the study is that the self-selection method of sampling meant that participants would not be representative of all TC patients. In particular, the sample may have included a disproportionate number of those who experienced difficulties during their treatment. The literature reports complication rates of 3.28% across all thyroidectomies,^62^ while eight of 25 patients in this study experienced postoperative complications. In addition, a previous study^63^ found that 45% of participants had no significant symptoms related to TC treatment, compared with only 4 of 25 in this study. Moreover, the study data collection took place in 2019 and 2020, incorporating the point when the global pandemic COVID-19 started. This could also impact people’s experience of TC treatment.

It was also not possible to implement the original research design, which would have involved collecting data from all participants through prospective longitudinal methods. However, the richness of data collected through the latter retrospective longitudinal approach suggests that this revision did not detrimentally affect the findings.

### Implications for practice and future research

Patients with TC should be treated in a kind and compassionate manner. It is not only an expectation but a requirement of conduct for all professionals working in the hospital and part of the values that all NHS employees should follow, even if they are not obliged to by a professional body. Questions should be encouraged, and the contact number of a person who can answer questions should be provided. Information for patients should be thorough and thyroid-specific. Patients should have access to information regarding support services and be referred when needed. All relevant referrals should be made when patients suffer from long-term physical or psychological symptoms. It is essential to acknowledge that the NHS may not be able to provide specialist counselling/support in all the areas discussed. For example, work and finances are specialist support areas, and the HCPs could most realistically make referrals to agencies that offer this type of specialist support. The areas regarding information provision, support, caring attitude of HCPs and hospital staff and managing side effects and symptoms from treatment are the areas that should be improved.

There are a number of questionnaires/inventories that seek to measure well-being. Among those that are most relevant to cancer patients are the Short-Form Health Survey-36, ThyPRO, European Organisation for Research and Treatment of Cancer Quality of Life Questionnaire Core 30 (EORTC QLQ-C30) and the Macmillan Concerns Checklist. For TC specifically, the MDASI-Thyroid Cancer module (MDASI-THY) has been used to demonstrate that fatigue, drowsiness, sleep disturbance, distress and difficulty remembering were the five most frequently experienced and severe symptoms of patients.^64^ The EORTC QLQ Thyroid Cancer Module (EORTC QLQ-THY34) is another inventory that has been shown to be a reliable and valid measure of the quality of life of TC patients.^65^ However useful these tools are, this study shows the value of conducting research which allows patients themselves to identify the issues that are most important to them. This approach could be extended to consider in more detail patients’ perspectives on specific interventions and their impact on well-being. Future research should investigate targeted interventions and evaluate their effectiveness in addressing the challenges faced by patients with TC.

### Conclusions

The findings of this study indicate that the well-being of TC patients is shaped by an interplay of treatment experiences, psychological impacts and the support they receive. Positive healthcare interactions and strong social support appeared to help participants cope more, whereas unmet information needs, long-term side effects and poor support experiences often added to distress. These results point to the potential value of strengthening patient education, emotional support and follow-up care. However, further work is required to determine the most effective strategies for meeting these needs in different healthcare contexts.

## Supplementary material

10.1136/bmjopen-2025-099254 online supplemental file 1

## Data Availability

No data are available.
