# Supplementary material for: Factors affecting the well-being of patients with thyroid cancer: results of a UK qualitative study
Source: BMJ Open. 2025 Oct 23;15(10):e099254. doi: 10.1136/bmjopen-2025-099254 (PMC12551542; doi:10.1136/bmjopen-2025-099254)
Supplement: online supplemental file 1 [file bmjopen-15-10-s001.docx]

| **Semi- Structured Interview Aide Memoire** |
| --- |
| **Project Title: Factors Affecting Wellbeing Among Thyroid Cancer Patients** |
| **Principal Investigator: Alicja Yilmaz**  **Example questions:**  **Before Treatment:** |

-Tell me about your life before the diagnosis

-Tell me how did you found out about thyroid cancer?

-Please can you describe how you felt when you received the thyroid cancer diagnosis?

-Please can you describe how you felt when you heard about the treatment that would be involved?

-Can you tell me about your experience of treatment to date?

-What did you think at that time?

-How did your friends and family react to your diagnosis, treatment?

-What are your arrangements for child care/work etc?

**During/After treatment: Above plus:**

-How did you cope with your family commitments? (After treatment begins)

-How did you cope with your work commitments?

-What was/has been the most difficult part of the process?

-What did you take most encouragement from?

-What was the biggest challenge in the cancer journey?

-What support did you have, wellbeing interventions etc?

-How did you cope with diagnosis/Treatment?

a. Surgery

b. RAI

**FOLLOW UP INTERVIEW GUIDE.**

The questions for the follow up will be guided by the answers provided by the participants in the first interview.

**HINTS**

**Experience questions:**

-Tell me more about that?

-What was it like for you?

-Can you describe an example?

**Feelings questions:**

-What did you think/feel at that time?

-How did it make you feel?

**Knowledge:**

-What treatment did you receive?

-Do you know how to access the services available to help you?

**Themes:**

-Feelings and thoughts

-Care received in hospital

-Coping with diagnosis

-Coping with symptoms

-Coping with surgery and RAI

-Family and friends

-Commitments (care, family, work)
