# Supplementary material for: Factors affecting the well-being of patients with thyroid cancer: results of a UK qualitative study
Source: BMJ Open. 2025 Oct 23;15(10):e099254. doi: 10.1136/bmjopen-2025-099254 (PMC12551542; doi:10.1136/bmjopen-2025-099254)
Supplement: Supplementary file 2 [file bmjopen-2025-099254.reviewer_comments.pdf]

PEER REVIEW HISTORY

BMJ Open publishes all reviews undertaken for accepted manuscripts. Reviewers are asked to complete a checklist review form and are provided with free text boxes to elaborate on their assessment. These free text comments are reproduced below.

ARTICLE DETAILS

Title (Provisional)

Factors affecting the well-being of thyroid cancer patients. Results of a UK qualitative study.

Authors

Rogusz, Alicja; Harding , Jamie

VERSION 1 - REVIEW

**Reviewer**	**1**
**Name**	**Parameswaran, Rajeev**
**Affiliation**	**Department of Endocrine Surgery, National University Hospital**
**Date**	**06-Jun-2025**
**COI**	**None**

Many thanks for the invitation to review the manuscript factors affecting the well-being of thyroid cancer patients - Results of a UK qualitative study. The authors have looked into very important aspects of cancer care which is often neglected especially in cancers with excellent prognosis. There are a lot of good aspects about the study and the premise of the study is quite good but there are significant issues with the study and need to be addressed.

Major points:

1. The numbers are very small to draw any conclusions, especially when the study was conducted at the COVID restrictions.

2. The current modalities of treatment also include active surveillance in carefully selected low risk well differentiated thyroid cancer, besides others as mentioned by the authors in the introduction.

3. Again, there is a paradigm shift in the management of well differentiated thyroid cancer based on the risk of recurrence. The very low risk and low risk papillary thyroid cancers do not need long term surveillance and are usually discharged from care after 2-3 years of follow up without any suppressive thyroxine therapy or radioiodine treatment.

4. Rephrase the statement The majority of the studies on the topic are quantitative (as the statement appears to be a broad claim from just 2 studies) to something like “Existing research on this topic has primarily relied on quantitative methods.

5. The authors claim they used extensive qualitative data but is not backed by the sample size.

6. In the objectives of the study, the authors mention about qualitative methods to explore the experiences of thyroid cancer patients – from diagnosis to recovery. Are the authors referring to medical or psychosocial recovery?

7. The authors in the also state one of the objectives as “to make recommendations for improvements to practice in the care of thyroid cancer patients.” This is a very strong statement to make from a small sample study. Perhaps would be better to amend the statement to something like “…to develop practice recommendations based on insights gained from patient narratives.”

8. In terms of setting of the study, the authors mention patients were recruited from NHS setting? Better to mention if they were recruited from outpatient surgical or endocrine clinics or any other?

9. There’s no mention of how recruitment adhered to ethical requirements, especially important when using charity mailing lists.

10. In the section of assessing rigour, GCP and GDPR are ethical and legal frameworks, not measures of trustworthiness (which relates to methodological integrity). This needs to be placed under ethical considerations. In the same section the authors mention that to aid credibility, the second researcher(JH) looked at some of the transcripts to ensure similar themes were identified to further ensure credibility (analyst triangulations). How were disagreements handled?

11. In the results section in Table 1, 22 patients were recruited from a non-NHS setting. In the methodology, the authors claim patients were recruited from a NHS setting. Kindly explain the discrepancy.

12. In table 3 of the results section, 3 patients had metastatic disease and should have been excluded from the analysis as the treatment and outcomes are very different to those with no metastasis. The higher stage of the disease will generally play a significant role in having more negative thoughts and poorer quality of life compared to those without metastasis. It is important to keep the cohort homogenous for a meaningful outcome analysis.

13. The authors describe that the most common negative factors include long-term physical symptoms including fatigue, psychological symptoms and post-operative symptoms. How many of the patients did have complications such as hypoparathyroidism or inadequate thyroxine replacement which may have contributed to their symptoms and hence poor quality of life.

14. One of the problems with healthcare and associated heterogeneity, especially in care from diagnosis to treatment and post therapy care. The authors need to include a few comments which ultimately may have an effect on their well being.

Minor:

1. The introductory line in the abstract is not correct. Thyroid cancer is a very common malignancy with low mortality compared to other human cancers. I believe the error may be a typo.

2. Interested patients contacted the researcher and PIS and consent form was sent. What is PIS?

3. “able to give inform consent. should read as informed consent

---

**Reviewer**	**2**
**Name**	**Carr, Alaina**
**Affiliation**	**Georgetown University**
**Date**	**18-Aug-2025**
**COI**	**None**

Abstract

In the background of the abstract, the authors state that existing studies assessing quality of life among TC populations are mainly quantitative. This is not an accurate statement.

Cho S, Tutelman P, Chomistek T, Rahamatullah I, Ryder-Burbidge C, Schulte F, et al. “No such thing as good cancer”: a qualitative exploration of the experience of early-onset thyroid cancer in survivors. Supportive Care in Cancer. 2024;32(10):671.

Randle RW, Bushman NM, Orne J, Balentine CJ, Wendt E, Saucke M, et al. Papillary thyroid cancer: the good and bad of the “good cancer.” Thyroid. 2017;27(7):902–7.

O'Neill CJ, et al. "Hearing the Voices of Australian Thyroid Cancer Survivors: Qualitative Thematic Analysis of Semistructured Interviews Identifies Unmet Support Needs." Thyroid 33.12 (2023): 1455-1464. Ovid MEDLINE(R). Web. 18 August. 2025. <http://ovidsp.ovid.com/ovidweb.cgi?T=JS&PAGE=reference&D=med24&NEWS=N&AN=37335225>.

Hsiao V, Hanlon BM, Robbins SE, Connor NP, Macdonald CL, Sippel RS, Schneider DF. Discovering domains important to health-related quality of life in differentiated thyroid cancer. The American Journal of Surgery. 2022 Oct 1;224(4):1176-8.

Kim K, et al. "Efficacy of a Home-Based Exercise Program After Thyroidectomy for Thyroid Cancer Patients." Thyroid 28.2 (2018): 236-245. Ovid MEDLINE(R). Web. 18 August. 2025.

<http://ovidsp.ovid.com/ovidweb.cgi?T=JS&PAGE=reference&D=med15&NEWS=N&AN=29258382>.

Introduction:

The authors state in the introduction lines 46-50 “The treatment includes partial hemithyroidectomy, total hemithyroidectomy and or RAI (Radioactive iodine treatment).” Total thyroidectomy, which is distinct from total hemithyroidectomy, is a primary treatment of thyroid cancer as well. Please include total thyroidectomy.

The side effects listed on lines 16-20, “Survivors of thyroid cancer may face lasting side effects and complications such as memory issues, headaches, emotional challenges, and symptoms associated with thyroid hormone suppressive therapy [9]” are from different treatments. Please elucidate the treatment-related side effects.

The authors state on lines 24-26. “The majority of the studies on the

topic are quantitative [15,16].” The authors state that existing studies assessing quality of life among TC populations are mainly quantitative. This is not accurate see citations provided above.

The authors state that the current study used “extensive qualitative data” (lines 29-32). In qualitative data analysis, a sample size of 25 is appropriate to reach data saturation, when no new themes emerge from the data. However, an N of 25 is not extensive qualitative data for qualitative data analysis. It is recommended that the authors rephrase this description.

Methods:

In the data collection subsection of the methods section, the authors state an interview guide was used. Please provide more detail about the type of interview guide. Was it a semi-structured interview guide? Who conducted the interviews?

In the Assessing Rigor subsection, how many transcripts did the second research review to ensure similar themes were identified?

What was the rationale for interviewing participants multiple times?

Results:

In Table 2 of the results section, the % for Gender do not add up to 100%. Please indicate whether there is missing data.

The authors should consider incorporating more quotes within the emerging themes to support the findings. For example, incorporating a patient quote following, “Moreover, many participants had anxiety regarding surgery, possible side effects, and about having scars on their neck” would provide further support for the emergence of this theme.

The authors state, “RAI treatment can be a positive or neutral experience for many participants.” Did participants in the sample express positive or neutral experiences? If so, indicate this sentiment is from the qualitative data.

Discussion:

Psychometrically valid assessment measures exist that assess quality of life among thyroid cancer populations. In particular, MDASI-Thy and QLQ-THY34 (see citations below). The SF-36 is a measure that does capture the statement made by the authors, “However, this study points to a need for a more comprehensive and holistic measurement tool, covering the range of medical, psychological and wider social factors that can affect wellbeing.” Please provide more clarity on the current gaps and how the results point to future research needs.

Gning I, Trask PC, Mendoza TR, Harle MT, Gutierrez KA, Kitaka SA et al. Development and initial validation of the thyroid cancer module of the M. D. Anderson symptom inventory. Oncology. 2008 Dec;76(1):59-68. doi: 10.1159/000178809

Singer S, Al-Ibraheem A, Pinto M, Iakovou I, Østhus AA, Hammerlid E, Locati LD, Gamper E, Ignacio J, Jordan SJ, Kiyota N, Buettner M, Engesser D, Canotilho R, Ioannidis G, Husson O, Gama RR, Fanetti G, Moss L, Inhestern J, Andry G, Fuehrer D, Kuliś D, Rimmele H, Sykiotis G. International Phase IV Field Study for the Reliability and Validity of the European Organisation for Research and Treatment of Cancer Thyroid Cancer Module EORTC QLQ-THY34. Thyroid. 2023 Sep;33(9):1078-1089. doi: 10.1089/thy.2023.0221. Epub 2023 Aug 9. PMID: 37450344.

Minor modifications:

Use acronyms consistently throughout the manuscript. For example, lines 50-51 TC is already defined earlier in the text but not included here, “The treatment journey of people with thyroid cancers can be quite complex and lengthy.”

The manuscript would benefit from additional proofreading and consistency with italicized quotations throughout the results section.

VERSION 1 - AUTHOR RESPONSE

**Reviewer 1:**	
1. The numbers are very small to draw any conclusions, especially when the study was conducted at the COVID restrictions.	The sample size is defended robustly in the Setting, Participants and Sampling section.
2. The current modalities of treatment also include active surveillance in carefully selected low risk well differentiated thyroid cancer, besides others as mentioned by the authors in the introduction.	We agree and have rewritten this section to clarify.
3. Again, there is a paradigm shift in the management of well differentiated thyroid cancer based on the risk of recurrence. The very low risk and low risk papillary thyroid cancers do not need long term surveillance and	We agree and have rewritten a sentence in the introduction accordingly:

are usually discharged from care after 2-3 years of follow up without any suppressive thyroxine therapy or radioiodine treatment.	"For this reason, some TC patients require ongoing follow-up for many years."
4. Rephrase the statement The majority of the studies on the topic are quantitative (as the statement appears to be a broad claim from just 2 studies) to something like "Existing research on this topic has primarily relied on quantitative methods.	Corrected and rephrased as suggested, thank you.
5. The authors claim they used extensive qualitative data but is not backed by the sample size.	As noted above, we have defended the sample size, which we believe provides substantial data. Word extensive has been deleted.
6. In the objectives of the study, the authors mention about qualitative methods to explore the experiences of thyroid cancer patients – from diagnosis to recovery. Are the authors referring to medical or psychosocial recovery?	The phrase 'to medical recovery and beyond' has been added to the relevant objective. Some retrospective interviews were conducted after medical recovery, but it is the assertion of this article that psychosocial issues could remain.
7. The authors in the also state one of the objectives as "to make recommendations for improvements to practice in the care of thyroid cancer patients." This is a very strong statement to make from a small sample study. Perhaps would be better to amend the statement to something like "...to develop practice recommendations based on insights gained from patient narratives."	Corrected, thank you for the suggestion.
8. In terms of setting of the study, the authors mention patients were recruited from NHS setting? Better to mention if they were recruited from outpatient surgical or endocrine clinics or any other?	Corrected. It is now stated that, where patients were recruited through the NHS, this was from a TC clinic.
9. There's no mention of how recruitment adhered to ethical requirements, especially important when using charity mailing lists. (charity social media which was included in ethical clearance)	It is made clear that the charity put out an appeal on social media and people who were

	interested in taking part contacted the research team. This approach had been ethically approved, as is indicated in the Settings, Participants and Sampling section.
10. In the section of assessing rigour, GCP and GDPR are ethical and legal frameworks, not measures of trustworthiness (which relates to methodological integrity). This needs to be placed under ethical considerations.	Corrected as suggested.
11. In the same section the authors mention that to aid credibility, the second researcher(JH) looked at some of the transcripts to ensure similar themes were identified to further ensure credibility (analyst triangulations). How were disagreements handled?	The Assuring Rigour section has been amended to incorporate this point.
12. In the results section in Table 1, 22 patients were recruited from a non-NHS setting. In the methodology, the authors claim patients were recruited from a NHS setting. Kindly explain the discrepancy.	The methodology section states that: *Patients were recruited from an NHS TC clinic setting, with the help of a local collaborator,* ***and*** *from the social media contact list of a relevant registered charity.*
13. In table 3 of the results section, 3 patients had metastatic disease and should have been excluded from the analysis as the treatment and outcomes are very different to those with no metastasis. The higher stage of the disease will generally play a significant role in having more negative thoughts and poorer quality of life compared to those without metastasis. It is important to keep the cohort homogenous for a meaningful outcome analysis.	The authors contest that it is right to include these three patients. The aim was to recruit a heterogeneous sample, representing a wide range of diagnoses and experiences of TC. The data did not support the idea that patients with metastatic disease feel more negative. Where there were differences between patients with different diagnoses and treatment regimes, these

	are highlighted in the discussion of the results.
14. The authors describe that the most common negative factors include long-term physical symptoms including fatigue, psychological symptoms and post-operative symptoms. How many of the patients did have complications such as hypoparathyroidism or inadequate thyroxine replacement which may have contributed to their symptoms and hence poor quality of life.	The findings indicate that 8 of 25 patients (32%) experienced post-operative complications and list those that were reported by patients. They were not prompted for specific complications/difficulties but asked to say for themselves what they regarded as a difficulty, which is consistent with the qualitative approach to the research and the desire to understand the patient perspective.
15. One of the problems with healthcare and associated heterogeneity, especially in care from diagnosis to treatment and post therapy care. The authors need to include a few comments which ultimately may have an effect on their well-being.	The framework used states that wellbeing includes negative and positive emotions and life satisfaction. Hence, all statements that deal with negative or positive emotions and assessment of situations have the effect on participants' wellbeing. Without participants necessarily talking about their wellbeing directly.<br><br>The impact on well-being is highlighted throughout the Results of Thematic Analysis and Discussion of Key Results sections.
**Minor:**	
1. The introductory line in the abstract is not correct. Thyroid cancer is a very common malignancy with low	The abstract was rewritten. The opening

mortality compared to other human cancers. I believe the error may be a typo.	paragraph of the Introduction clarifies this issue.
2. Interested patients contacted the researcher and PIS and consent form was sent. What is PIS? (Participant Information Sheet)	Yes, it is. This abbreviation has been explained at the first mention.
3. "able to give inform consent. should read as informed consent.	Corrected.
**Reviewer 2:**	
Abstract<br>1. In the background of the abstract, the authors state that existing studies assessing quality of life among TC populations are mainly quantitative. This is not an accurate statement.<br><br>Cho S, Tutelman P, Chomistek T, Rahamatullah I, Ryder-Burbidge C, Schulte F, et al. "No such thing as good cancer": a qualitative exploration of the experience of early-onset thyroid cancer in survivors. Supportive Care in Cancer. 2024;32(10):671.<br><br>Randle RW, Bushman NM, Orne J, Balentine CJ, Wendt E, Saucke M, et al. Papillary thyroid cancer: the good and bad of the "good cancer." Thyroid. 2017;27(7):902–7.<br><br>O'Neill CJ, et al. "Hearing the Voices of Australian Thyroid Cancer Survivors: Qualitative Thematic Analysis of Semistructured Interviews Identifies Unmet Support Needs." Thyroid 33.12 (2023): 1455-1464. Ovid MEDLINE(R). Web. 18 August. 2025. .<br><br>Hsiao V, Hanlon BM, Robbins SE, Connor NP, Macdonald CL, Sippel RS, Schneider DF. Discovering domains important to health-related quality of life in differentiated thyroid cancer. The American Journal of Surgery. 2022 Oct 1;224(4):1176-8.<br><br>Kim K, et al. "Efficacy of a Home-Based Exercise Program After Thyroidectomy for Thyroid Cancer Patients." Thyroid 28.2 (2018): 236-245. Ovid MEDLINE(R). Web. 18 August. 2025. .	We are grateful that these studies have been brought to our attention. They are referred to in the introduction, together with a note that ours is the first UK study of thyroid cancer patients to use predominantly qualitative data.

Introduction:<br>2. The authors state in the introduction lines 46-50 "The treatment includes partial hemithyroidectomy, total hemithyroidectomy and or RAI (Radioactive iodine treatment)." Total thyroidectomy, which is distinct from total hemithyroidectomy, is a primary treatment of thyroid cancer as well. Please include total thyroidectomy.	Corrected and slightly rewritten.
3.The side effects listed on lines 16-20, "Survivors of thyroid cancer may face lasting side effects and complications such as memory issues, headaches, emotional challenges, and symptoms associated with thyroid hormone suppressive therapy [9]" are from different treatments. Please elucidate the treatment-related side effects.	The section rewritten
4.The authors state on lines 24-26. "The majority of the studies on the<br>topic are quantitative [15,16]." The authors state that existing studies assessing quality of life among TC populations are mainly quantitative. This is not accurate see citations provided above.	As noted above, the relevant studies are now cited in the introduction. Section rewritten.
5.The authors state that the current study used "extensive qualitative data" (lines 29-32). In qualitative data analysis, a sample size of 25 is appropriate to reach data saturation, when no new themes emerge from the data. However, an N of 25 is not extensive qualitative data for qualitative data analysis. It is recommended that the authors rephrase this description.	The word extensive was deleted from the manuscript. The issue of sample size is dealt with above and in **Setting, participants and sampling** section.
Methods:<br>6. In the data collection subsection of the methods section, the authors state an interview guide was used. Please provide more detail about the type of interview guide. Was it a semi-structured interview guide? Who conducted the interviews?	The Data Collection section has been amended to say that interviews were semi-structured and were conducted by Alicja Rogusz.
7. In the Assessing Rigor subsection, how many transcripts did the second research review to ensure similar themes were identified?	It is now stated that five transcripts were reviewed.
8.What was the rationale for interviewing participants multiple times?	This is explained in a new paragraph of the Data Collection section (the final paragraph),

Results:<br>9. In Table 2 of the results section, the % for Gender do not add up to 100%. Please indicate whether there is missing data.	This was an error and has been corrected to 88% and 12%.
10.The authors should consider incorporating more quotes within the emerging themes to support the findings. For example, incorporating a patient quote following, "Moreover, many participants had anxiety regarding surgery, possible side effects, and about having scars on their neck" would provide further support for the emergence of this theme.	Five quotations have been added to further illustrate themes, including one to add detail to this point about anxiety.
11.The authors state, "RAI treatment can be a positive or neutral experience for many participants." Did participants in the sample express positive or neutral experiences? If so, indicate this sentiment is from the qualitative data.	This has been changed to: 'RAI treatment had been a positive or neutral experience for many participants interviewed in the study.'
Discussion:<br>12.Psychometrically valid assessment measures exist that assess quality of life among thyroid cancer populations. In particular, MDASI-Thy and QLQ-THY34 (see citations below). The SF-36 is a measure that does capture the statement made by the authors, "However, this study points to a need for a more comprehensive and holistic measurement tool, covering the range of medical, psychological and wider social factors that can affect wellbeing." Please provide more clarity on the current gaps and how the results point to future research needs.<br><br>Gning I, Trask PC, Mendoza TR, Harle MT, Gutierrez KA, Kitaka SA et al. Development and initial validation of the thyroid cancer module of the M. D. Anderson symptom inventory. Oncology. 2008 Dec;76(1):59-68. doi: 10.1159/000178809<br><br>Singer S, Al-Ibraheem A, Pinto M, Iakovou I, Østhus AA, Hammerlid E, Locati LD, Gamper E, Ignacio J, Jordan SJ, Kiyota N, Buettner M, Engesser D, Canotilho R, Ioannidis G, Husson O, Gama RR, Fanetti G, Moss L, Inhestern J, Andry G, Fuehrer D, Kuliś D, Rimmele H, Sykiotis G. International Phase IV Field Study for the Reliability and	Again, we are grateful for bringing our attention to these scales, which have been incorporated into the second paragraph of the Implications for Practice and Future Research section.

Validity of the European Organisation for Research and Treatment of Cancer Thyroid Cancer Module EORTC QLQ-THY34. Thyroid. 2023 Sep;33(9):1078-1089. doi: 10.1089/thy.2023.0221. Epub 2023 Aug 9. PMID: 37450344.	
**Minor modifications:**	
1. Use acronyms consistently throughout the manuscript. For example, lines 50-51 TC is already defined earlier in the text but not included here, "The treatment journey of people with thyroid cancers can be quite complex and lengthy."	Corrected for Thyroid Cancer (TC), Radioactive Iodine (RAI), Butterfly Thyroid Cancer Trust (BTCT), Partial Thyroidectomy (PT), Total Thyroidectomy (TT), Participant Information Sheet (PIS)
2. The manuscript would benefit from additional proofreading and consistency with *italicized* quotations throughout the results section.	Both authors have completed thorough proofreading. All quotations *italicised*
